# Analysis on the Filament Structure Evolution in Reset Transition of Cu/HfO_2_/Pt RRAM Device

**DOI:** 10.1186/s11671-016-1484-8

**Published:** 2016-05-25

**Authors:** Meiyun Zhang, Shibing Long, Yang Li, Qi Liu, Hangbing Lv, Enrique Miranda, Jordi Suñé, Ming Liu

**Affiliations:** Key Laboratory of Microelectronics Devices and Integrated Technology, Institute of Microelectronics of Chinese Academy of Sciences, Beijing, 100029 China; Jiangsu National Synergetic Innovation Center for Advanced Materials (SICAM), Nanjing, 210023 China; Departament d’Enginyeria Electrònica, Universitat Autònoma de Barcelona, Bellaterra, 08193 Spain

**Keywords:** RRAM, Conductive filament (CF), Structure evolution, Monte Carlo simulator

## Abstract

The resistive switching (RS) process of resistive random access memory (RRAM) is dynamically correlated with the evolution process of conductive path or conductive filament (CF) during its breakdown (rupture) and recovery (reformation). In this study, a statistical evaluation method is developed to analyze the filament structure evolution process in the reset operation of Cu/HfO_2_/Pt RRAM device. This method is based on a specific functional relationship between the Weibull slopes of reset parameters’ distributions and the CF resistance (*R*_on_). The CF of the Cu/HfO_2_/Pt device is demonstrated to be ruptured abruptly, and the CF structure of the device has completely degraded in the reset point. Since no intermediate states are generated in the abrupt reset process, it is quite favorable for the reliable and stable one-bit operation in RRAM device. Finally, on the basis of the cell-based analytical thermal dissolution model, a Monte Carlo (MC) simulation is implemented to further verify the experimental results. This work provides inspiration for RRAM reliability and performance design to put RRAM into practical application.

## Background

With conventional flash memories approaching their technical and physical limits, there will be severe problems in the scaling of solid-state memory [1–4]. A great amount of research attention has been focused on the next generation memory devices. Resistive random access memory (RRAM), with the reversible and reproducible resistive switching (RS) phenomena induced by applied electric field has been extensively studied due to its potential applications in high density memory [5] and neuromorphic electronic systems [6–9]. The electrochemical metallization (ECM)-based RRAM with an active metal electrode such as Ag or Cu is referred to as programmable metallization cell (PMC) or conductive bridge RAM (CBRAM), which is an important type of RRAM device. The RS phenomena of the PMC are attributed to the oxidation of the active anode metal into cations, the transport of these cations, and their reduction on the cathode or in the RS layer [10–12]. Via the above redox process, the nanoscale conductive filaments (CFs) are formed in the set process and ruptured in the reset process in the RS layer [13–21]. The confinement of the resistive switching phenomenon to a nanometric filament has been widely demonstrated by conductive AFM [22–24] and cross-sectional TEM [25, 26]. However, the filamentary switching has the stochastic nature, similar to the dielectric breakdown, which has ever been a serious obstacle to boost RRAM into practical applications [27–30]. Studying the statistics of the RS parameters [31, 32] is also significant to discover the filament annihilation/reconstruction information and guide us to improve the uniformity. The Weibull distribution has been often used to analyze the statistics of electron devices. Because the initial filament width or on-state resistance (*R*_on_) has a significant impact on the reset transition process and there is an analytical correlationship between the Weibull slopes (*β*) of reset parameters’ distributions and CF size or *R*_on_ [33, 34], this relationship could be made use of to analyze the filament microstructure evolution. At the reset point where the current is the maximum in the reset *I*–*V* curve, when *β* the Weibull slope changes with *R*_on_, the degradation of the CF structure has occurred, and the reset transition inclines to be abrupt [33]. On the contrary, the CF just starts to dissolve at the reset point and the reset switching tends to be gradual [34] when *β* the Weibull slope is a constant, independent on *R*_on_.

In this paper, the annihilation behavior of the filament in Cu/HfO_2_/Pt PMC device is investigated according to the above mentioned statistical evaluation method. The Weibull slopes (*β*_*V*_ and *β*_*I*_) of our Cu/HfO_2_/Pt CBRAM device decrease with *R*_on_, so the filament dissolution or the reset transition is abrupt. A Monte Carlo method is utilized to simulate and capture the experimental results. The controllable abrupt reset operation will bring great benefits to the reliable binary operation of RRAM. Our work has great significance in providing inspiration for RRAM performance and reliability design to put RRAM into practical application.

## Methods

**Fig. 1:**
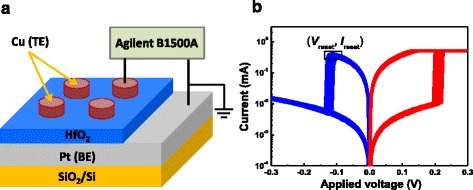
**a** Structure of the Cu/HfO_2_/Pt RRAM device. **b** *I*–*V* curves of Cu/HfO_2_/Pt RRAM device under the compliance current of 500 μA

The Cu/HfO_2_/Pt device with the schematic structure shown in Fig. 1a is comprised of an inert Pt bottom electrode (BE), a HfO_2_ RS layer, and an oxidizable Cu metal top electrode (TE). A 20-nm-thick Pt BE and a 10-nm-thick HfO_2_ layer were sequentially deposited by magnetron sputtering on SiO_2_/Si substrate. Then, Cu TE was sputtered and patterned to have a thickness of 40 nm and an area of 100 × 100 μm^2^. The electrical characteristics of the device were measured by Agilent B1500A semiconductor device parameter analyzer. The *I*–*V* curves were tested under the DC voltage sweep mode, where the bias voltage was applied to the TE with the BE grounded. Figure 1b shows the 20 *I*–*V* curves of the Cu/HfO_2_/Pt device.

## Results and Discussion

**Fig. 2:**
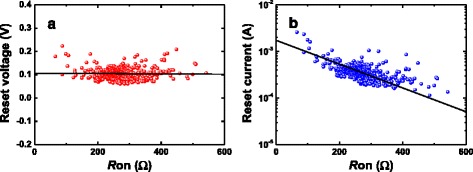
**a** The scatter plot of *V*_reset_ of Cu/HfO_2_/Pt device as a function of *R*_on_. The *straight line* is the fitting line. *V*_reset_ is independent of *R*_on_. **b** The scatter plot of *I*_reset_ of Cu/HfO_2_/Pt device as a function of *R*_on_. The *straight line* is the fitting line. *I*_reset_ decreases with *R*_on_

Figure 1b shows 20 *I*–*V* curves of the Cu/HfO_2_/Pt device. We can find that these curves present abrupt switching during set and reset cycles. The reset points are defined as those having and are the maximum current in the *I*–*V* curves in reset process, and their voltages and currents are defined as *V*_reset_ and *I*_reset_, respectively. To investigate whether the degradation of CF microstructure has occurred or not before the reset point, 1000 continuous set/reset cycles have been measured to get the *V*_reset_ and *I*_reset_ statistical characteristics. Figure 2 presents the scatter plots for *V*_reset_ and *I*_reset_ dependent on *R*_on_. *V*_reset_ keeps constant and *I*_reset_ decreases with *R*_on_. We can find that *R*_on_ has influence on some parameters of *V*_reset_ and *I*_reset_ distributions.

**Fig. 3:**
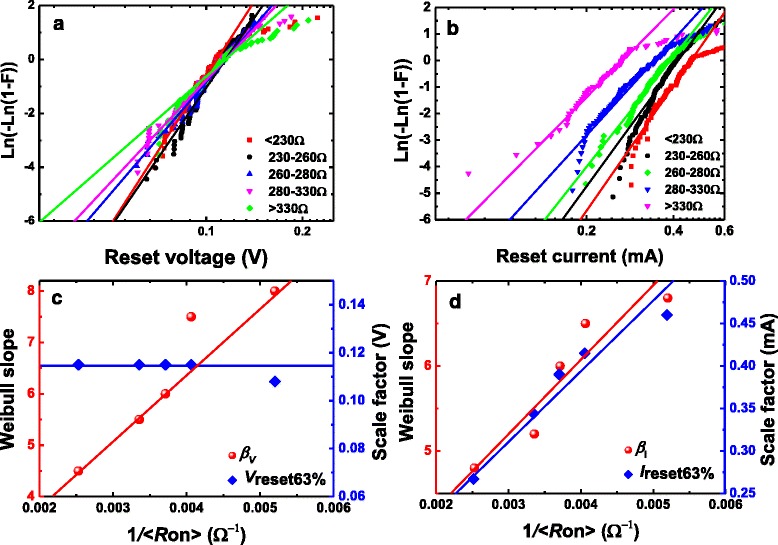
The distributions of *V*_reset_ (**a**) and *I*_reset_ (**b**) of Cu/HfO_2_/Pt RRAM device in different *R*_on_ groups. The *straight lines* are those of fitting to the standard Weibull distribution. **c** The dependence of Weibull slope (*β*_*V*_) and scale factor (*V*_reset63%_) of *V*_reset_ distributions on 1/*R*_on_. *β*_*V*_ the Weibull slope and 1/*R*_on_ have a linear relation while *V*_reset63%_ the scale factor keeps constant. **d** The dependence of Weibull slope (*β*_*I*_) and scale factor (*I*_reset63%_) of *I*_reset_ distributions on 1/*R*_on_. Both *β*_*I*_ and *I*_reset63%_ are in linear to 1/*R*_on_

**Fig. 4:**
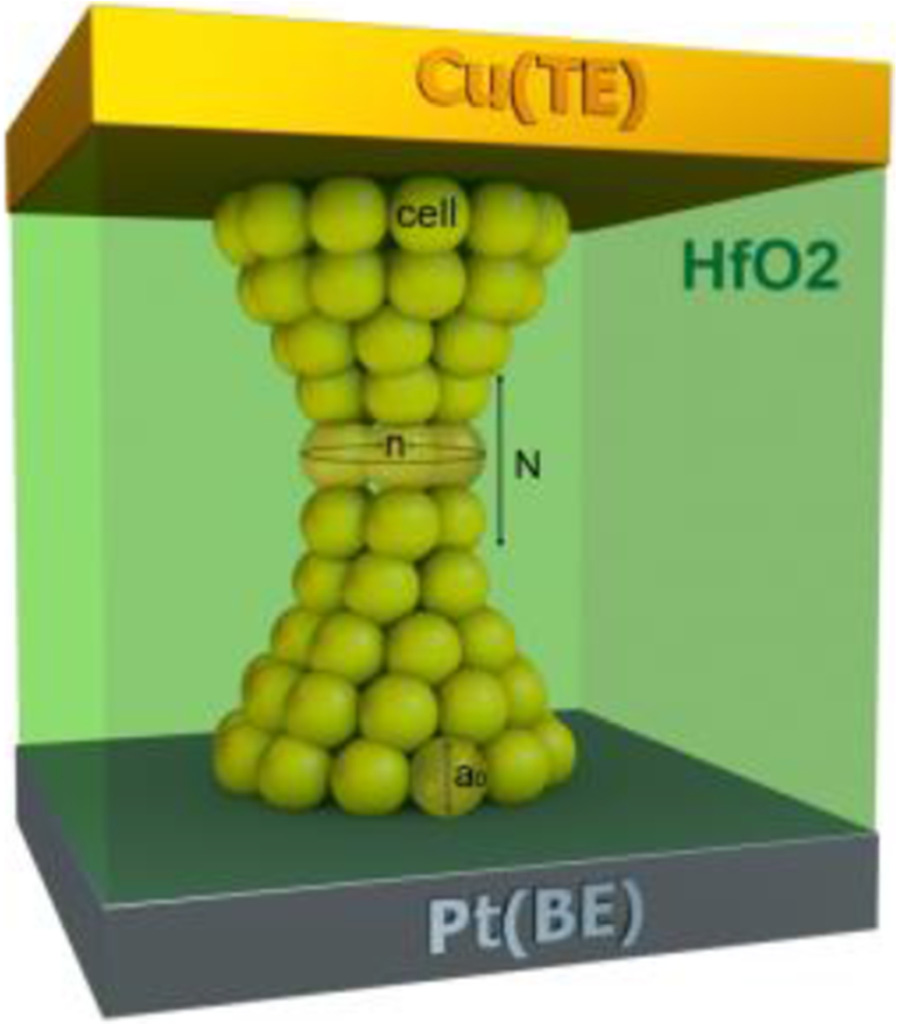
Schematic of the cell-based model of the CF in the RS layer. *N* is the number of slices (CF length) of the most constrictive part of the CF and *n* is the number of cells in each slice (CF width)

To study the correlation of *V*_reset_ and *I*_reset_ with *R*_on_ in detail, the whole *R*_on_ range was divided into several ranges using the screening method [33, 34]. The method of the separation of the data into different groups does not influence the final statistical results, i.e., the results keep a certain regularity regardless of the different grouping methods. Weibull distribution is used to describe the distributions of *V*_reset_ and *I*_reset_ in each range. Figure 3a, b shows the Weibull distributions of *V*_reset_ and *I*_reset_ in grouped *R*_on_ range, respectively. We can find that the distributions in each range have some tails. However, these tails just occupy a little proportion of the overall distribution in each range, which does not affect the global tendency of the distribution. Through the linear fittings to experimental *V*_reset_ and *I*_reset_ distributions in different groups, we can obtain the Weibull slopes (*β*_*V*_ and *β*_*I*_) and scale factors (*V*_reset63%_ and *I*_reset63%_). Figure 3c, d shows that *β*_*V*_ and *β*_*I*_ Weibull slopes are linear to 1/*R*_on_, while *V*_reset63%_ the scale factor is constant and *I*_reset63%_ is linear to 1/*R*_on_. The experimental results can be explained by the cell-based thermal dissolution model [33] with its geometric model shown in Fig. 4. According to this model, the reset is determined by the narrowest part of the filament consisting of *N* slices of cells with each slice including *n* cells. When at least one slice of cells is “defective” under thermal dissolution mechanism, e.g., the oxygen vacancies are occupied by oxygen ions, the reset transition occurs. In Ref. [33], the cell model was constructed for unipolar valence change mechanism (VCM) device in which the reset transition is dominated by the thermal dissolution of CF. Here we find that the cell model is also suitable for the experimental statistics of oxide-based ECM device in this work. The reset of this kind of ECM device can be understood as that the metal atoms (Cu) in CF are oxidized into cations and diffuse out from the CF region under the Joule heat generated in CF. The most important result of the cell model is that the Weibull slopes of *V*_reset_ and *I*_reset_ distribution are linearly dependent on the CF size, i.e., 1/*R*_on_, which is expressed by:

1 $$ {\beta}_V={\beta}_I=kn, $$

where *n* = *R*_0_/*R*_on_, *R*_0_ is the resistance value of a single CF path with one chain of cells and *k* is a parameter related to the defect generation and diffusion [33]. The Weibull slope proportional to *n*, i.e., 1/*R*_on_ in Eq. (1), indicates that under the thermal dissolution effect [34], defects in the cells have diffused out and the constrictive part of the CF has changed before the reset point. Thus, according to the model, the change of Weibull slopes of *V*_reset_ and *I*_reset_ distributions as a function of *R*_on_ in the Cu/HfO_2_/Pt RRAM device in this work indicates that the microstructure of the CF has degraded under the thermal dissolution effect. In a previous study [34], the Weibull slopes of the switching parameters independent of the initial resistance state indicate that the reset point corresponds to the initial step in CF dissolution. The abrupt or gradual reset transition is closely related to the initial CF resistance (*R*_on_), according to the thermal dissolution mechanism [35], which can be influenced by the current compliance during the measurement. The drastic dissolution of the CF may be attributed to a great deal of Joule heat produced in the stronger CF with lower *R*_on_ and the heat loss along the CF [35]. The analytical cell-based reset model can provide an inspiration for the analysis of what has happened in the CF of Cu/HfO_2_/Pt RRAM before the reset point.

To better interpret and simulate the experimental reset statistics of the Cu/HfO_2_/Pt device, a Monte Carlo simulator has been established based on the proposed cell-based model for the reset statistics [33]. In our simulation, *V*_reset_ is assumed as to present an arbitrary Weibull distribution and is assumed as expressed by:

2 $$ {V}_{\mathrm{reset}}={V}_{\mathrm{reset}63\%}\mathrm{L}\mathrm{n}{\left(1-{F}_V\right)}^{1/{\beta}_V}, $$

where *F*_*V*_ = *r*_1_, *n* = *n*_min_ + (*n*_max_ − *n*_min_)*r*_2_. *V*_reset63 %_ is the scale factor abstracted from the experimental global *V*_reset_ distribution and *r*_1_ and *r*_*2*_ are random numbers between 0 and 1. Using Eqs. (1) and (2), the simulated *I*_reset_ distributions can be obtained by:

3 $$ {I}_{\mathrm{reset}}={V}_{\mathrm{reset}}/{R}_{\mathrm{on}}. $$

**Fig. 5:**
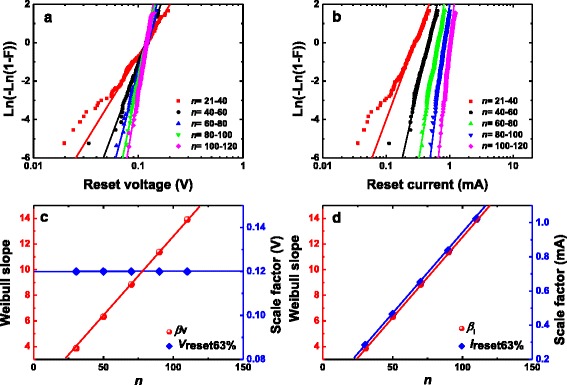
The MC-simulated Weibull distributions of *V*_reset_ (**a**) and *I*_reset_ (**b**) in different *n* groups. The *straight lines* are fitting lines. **c** The dependence of the MC-simulated *β*_*V*_ and *V*_reset63%_ on *n. β*_*V*_ the Weibull slope and *n* have a linear relation while *V*_reset63%_ the scale factor keeps constant. **d** The dependence of the MC-simulated *β*_*I*_ and *I*_reset63%_ Weibull slope and scale factor as a function of *n*. Both of them increase with *n* linearly

In the simulation, we use *V*_reset 63%_ = 0.12 V on the basis of the experimental result in Fig. 3a. Since *R*_0_ represents the resistance of a single CF path with one chain of cells, we can assume *R*_0_ = 1/*G*_0_, where *G*_0_ = 2*e*^2^/*h* is the quantum of conductance, as we have adopted in Ref. [36]. According to the range of *R*_on_ in Fig. 3, we can calculate that *n*_min_ = 21 and *n*_max_ = 120. By fitting the experimental *β*–1/*R*_on_ data in Fig. 3c, d with Eq. (1), *k* = 0.124 can be got. The above values are used to conduct the simulation. One thousand cycles have been constructed to match the practical number of experimental switching cycles. For each cycle, according to Eqs. (2) and (3), the simulated *V*_reset_ and *I*_reset_ values were achieved through generating random values for *r*_1_ and *r*_2_. Then we study the statistical distribution of the simulated *V*_reset_ and *I*_reset_ in each *n* group. Figure 5a, b illustrates the simulated *V*_reset_ and *I*_reset_ distributions in each *n* range. Figure 5c, d presents the Weibull slopes of *V*_reset_ and *I*_reset_ which have a linear correlation with *n*, i.e., linearly increase with or 1/*R*_on_ and the scale factor of *V*_reset_ is independent of *R*_on_ while that of *I*_reset_ increases with 1/*R*_on_ in linearity. The simulated results perfectly capture the experimental results. Thus, the dissolution event has finished in the CF in the reset point, which is demonstrated from both the experimental and simulation aspects.

As the abrupt reset behavior has the advantages to the reliable binary operation of RRAM, it is important to control the reset transition. Some methods can be used to get the abrupt reset switching. For example, utilizing current sweep [37, 38] operation in a single RRAM cell or using gate voltage sweep operation in a 1T1R structure [39], the reset transition can be implemented to preset well-controlled abrupt switching characteristics. By combining the above method with the approaches of increasing resistances such as introducing a barrier layer, it is expected to achieve the abrupt and low-power set/reset operation.

## Conclusions

The detailed microstructure evolution before the reset point in the CF of Cu/HfO_2_/Pt RRAM devices has been analyzed. The Weibull slopes of our device change with the different on-resistance or CF size. This result indicates that dissolution has just finished at the reset point. The obvious Joule heat generation in the wide CF may be the underlying reason for the drastic CF dissolution. To model the experimental results, a Monte Carlo simulator has been established and the simulated results are fully in consistency with those of the experiment.
